# A prognostic signature established based on genes related to tumor microenvironment for patients with hepatocellular carcinoma

**DOI:** 10.18632/aging.205722

**Published:** 2024-04-04

**Authors:** Zhongfeng Cui, Ge Li, Yanbin Shi, Xiaoli Zhao, Juan Wang, Shanlei Hu, Chunguang Chen, Guangming Li

**Affiliations:** 1 Department of Clinical Laboratory, Henan Provincial Infectious Disease Hospital, Zhengzhou 450000, China; 2 Department of Radiology, Henan Provincial Infectious Disease Hospital, Zhengzhou 450000, China; 3 Department of Infectious Diseases and Hepatology, Henan Provincial Infectious Disease Hospital, Zhengzhou 450000, China

**Keywords:** tumor microenvironment, hepatocellular carcinoma, molecular subtypes, risk score model, prognosis

## Abstract

Background: Complex cellular signaling network in the tumor microenvironment (TME) could serve as an indicator for the prognostic classification of hepatocellular carcinoma (HCC) patients.

Methods: Univariate Cox regression analysis was performed to screen prognosis-related TME-related genes (TRGs), based on which HCC samples were clustered by running non-negative matrix factorization (NMF) algorithm. Furthermore, the correlation between different molecular HCC subtypes and immune cell infiltration level was analyzed. Finally, a risk score (RS) model was established by LASSO and Cox regression analyses (CRA) using these TRGs. Functional enrichment analysis was performed using gene set enrichment analysis (GSEA).

Results: HCC patients were divided into three molecular subtypes (C1, C2, and C3) based on 704 prognosis-related TRGs. HCC subtype C1 had significantly better OS than C2 and C3. We selected 13 TRGs to construct the RS model. Univariate and multivariate CRA showed that the RS could independently predict patients’ prognosis. A nomogram integrating the RS and clinicopathologic features of the patients was further created. We also validated the reliability of the model according to the area under the receiver operating characteristic (ROC) curve value, concordance index (C-index), and decision curve analysis. The current findings demonstrated that the RS was significantly correlated with CD8+ T cells, monocytic lineage, and myeloid dendritic cells.

Conclusion: This study provided TRGs to help classify patients with HCC and predict their prognoses, contributing to personalized treatments for patients with HCC.

## INTRODUCTION

The liver is an immunological organ that harbors abundant immune cells such as dendritic cells (DCs), natural killer (NK) cells, and gamma delta (γ δ) T cells, which will trigger immune response to protect the body against adverse pathogens and prevent tumorigenesis [1]. HCC as the most common subtype of liver cancer has the third highest death rate [2]. Unlike normal liver, the immunosuppressive TME of patients is characterized by the presence of immune cells, for example, macrophages, myeloid-derived suppressor cells, tumor-correlated neutrophils, regulatory T cells, lymphocytes infiltrating tumors, and CD8+ cytotoxic T lymphocytes and tumor vasculature [3, 4]. Previous studies have explored the TME in the biological and prognostic classification of patients with HCC [5] and proposed an RME-based classification for HCC patients according to the mRNA and protein expression detected by multiplex-immunohistochemistry (IHC) and mass cytometry (CyTOF) [6]. Patients with immunosuppressive TME, including high levels of regulatory T cells (Tregs) and myeloid-derived suppressor cells (MDSCs), will often develop poorer prognosis and benefit limitedly from conventional and immunotherapeutic treatments. Conversely, TME enriched with active immune components such as CD8+ T cells often indicates a favorable prognosis and better responses to certain therapies [7–10]. Therefore, the heterogeneity of TME and patients’ immune status should be comprehensively explored in order to improve the clinical treatment of HCC.

TME is a complex network of signaling pathways as it contains a variety of cell types, including cancer, stromal, endothelial, and immune cells, and non-cellular components such as growth factors and cytokines [11]. Currently, targeting the TME as an option to treat patients with HCC is still difficult [12], which also points to the need to characterize the TME of patients with HCC and identify TME-related molecular signatures [5].

This study excavated TME-related genes (TRGs) in patients with HCC and developed a risk score (RS) model using these TRGs. Samples were clustered using TRGs and non-negative matrix factorization (NMF) algorithm. The RS model was established and validated by performing LASSO and Cox regression analysis (CRA). The present study developed a TME-based classification for HCC patients and filtered TME-related markers for prognostic prediction, contributing to the personalized treatment in HCC.

## MATERIALS AND METHODS

### Data acquisition and preprocessing

We collected the transcriptomic data and clinical features of 374 HCC patients and 50 control samples from The Cancer Genome Atlas (TCGA) database by visiting Genomic Data Commons (https://portal.gdc.cancer.gov/) [13]. Four patients without complete clinical follow-up and microdissection data were eliminated, while the remaining samples were included in the analyses. Moreover, for external validation, the GSE76427 dataset, which used the GPL10558 platform and included 115 primary HCC tumors and 52 adjacent nontumor tissues, were obtained from the Gene Expression Omnibus (GEO) database (https://www.ncbi.nlm.nih.gov/geo/).

### Screening and analysis of differentially expressed TRGs (DE-TRGs)

We identified 4,061 TRGs from previously published studies [14, 15]. First, “limma” package was employed to screen DE-TRGs between HCC and adjacent nontumor tissues [16]. The data in fpkm format were preprocessed by normalization and filtering under the criteria of the absolute value of FDR <0.05 and |log2Fold Change|>1.

### NMF clustering

Prognosis-related DE-TRGs were screened using Univariate CRA (UCRA) under *P* < 0.01 as the threshold. Next, NMF clustering analysis was performed on the expression matrix of prognosis-related DE-TRGs in the “NMF” R package [17]. The correlation coefficients were calculated, the inner feature structure of gene expression matrices was predicted, and 50 iterations were recycled during the clustering.

### Analysis of immune cell infiltration

Microenvironment cell populations-counter (MCP-counter) was applied to score the abundance of immune cells such as CD8+ T cells, NK and endothelial cells, myeloid DCs, neutrophils, cytotoxic and B lymphocytes, monocyte-derived cells, and fibroblasts based on the gene expression [18] using the “MCPcounter” package.

### Developing and validating the RS model

We divided 370 HCC patients from TCGA into the training (*n* = 262) and validation (*n* = 108) sets at the ratio of 7:3 ratio without control (Table 1). Notably, the GSE76427 dataset contained both tumor and adjacent non-tumor samples, only the tumor samples were used for developing a RS model. Next, prognosis-related genes were mined by performing UCRA on DE-TRGs, the model was refined by building a penalized feature using the “glmnet” package [19], and multivariate CRA (MCRA) was used to screen the characteristic genes for the RS model. Finally, we calculated the coefficient value of each gene and the riskscore as follows:


$$Riskscore=\sum_{i=1}^{n}\left(coefi\times Xi\right)$$

*coef* represented the Cox regression coefficient for each gene, and X referred to gene expression.

**Table 1:** 370 sample information about training set and verification set of TCGA-LIHC.

**Characteristics**	**TCGA-LIHC cohort (*N* = 370)**	**GSE76427 (*N* = 115)**
**Age**		
Mean ± SD	59.45 ± 13.51	63.45 ± 12.68
Median (min-max)	61.00 (16.00, 90.00)	64.00 (14.00, 93.00)
**Gender**		
Female	121 (32.7%)	22 (19.13%)
Male	249 (67.3%)	93 (80.87%)
**Grade**		
G1	55 (14.86%)	NA
G2	177 (47.84%)	NA
G3	121 (32.7%)	NA
G4	12 (3.24%)	NA
Unknow	5 (1.35%)	NA
**Stage**		
Stage I	171 (46.22%)	55 (47.83%)
Stage II	85 (22.97%)	35 (30.43%)
Stage III	85 (22.97%)	21 (18.26%)
Stage IV	5 (1.35%)	4 (3.48%)
Unknow	24 (6.49%)	NA
**T**		
T1	181 (48.92%)	NA
T2	93 (25.14%)	NA
T3	80 (21.62%)	NA
T4	13 (3.51%)	NA
TX	1 (0.27%)	NA
Unknow	2 (0.54%)	NA
**M**		
M0	345 (93.24%)	NA
M1	4 (1.08%)	NA
MX	21 (5.68%)	NA
**N**		
N0	329 (88.92%)	NA
N1	4 (1.08%)	NA
NX	37(10%)	NA
**Survival status**		
Dead	240 (64.86%)	92 (80.00%)
Alive	130 (35.14%)	23 (20.00%)

Abbreviation: NA: Not Available.

The samples were clustered by their median RS value into the low-risk group (LRG) and high-risk group (HRG). The prognostic significance of the model was analyzed by Kaplan-Meier (KM) analysis with two-sided log-rank test using the “survminer” package. Area under the receiver operating characteristic (ROC) curve (AUC) in “timeROC” [20] package was used to reflect the prediction accuracy of the model.

We also created a nomogram by integrating clinicopathological features such as gender, age, TNM stages, grade, clinical stage, and the RS model using the “restricted mean survival (rms)” package. The reliability of the nomogram was tested according to the ROC curve, calibration curve (CC, bootstrap 1000), decision curve analysis (DCA), and C-index.

### Analysis of biological functions

Annotated gene sets ‘c2.cp.kegg.v7.4’ and ‘c5.go.v7.4.symbols’ obtained from the Molecular Signatures Database (MSigDB, http://www.gsea-msigdb.org/gsea/msigdb) were used for GSEA (software version 4.1.0).

### Statistical analysis

Data were analyzed using the R (version 4.1.1) package. Volcano maps and violin plots were visualized by the “ggplot2” package [21] and “ggpubr” package, respectively. CRA was performed using the “survival” package. By employing the chi-squared or Fisher’s exact test, differences between both sets and the correlation between clinical characteristics and the RS were analyzed. KM survival curves with the log-rank tests were generated using the “survminer” package. The ROC and calibration curves were plotted using the “timeROC” and “rms” packages, respectively [22]. The restricted mean survival (RMS) package was used for computing the C-index of the model. A *p* < 0.05 indicated a significant difference.

### Data availability statement

The dataset analyzed in this study is available in (GSE76427) at (https://www.ncbi.nlm.nih.gov/geo/query/acc.cgi?acc=GSE76427).

## RESULTS

### NMF consensus clustering analysis classified three molecular HCC subtypes based on TRGs

**Figure 1:**
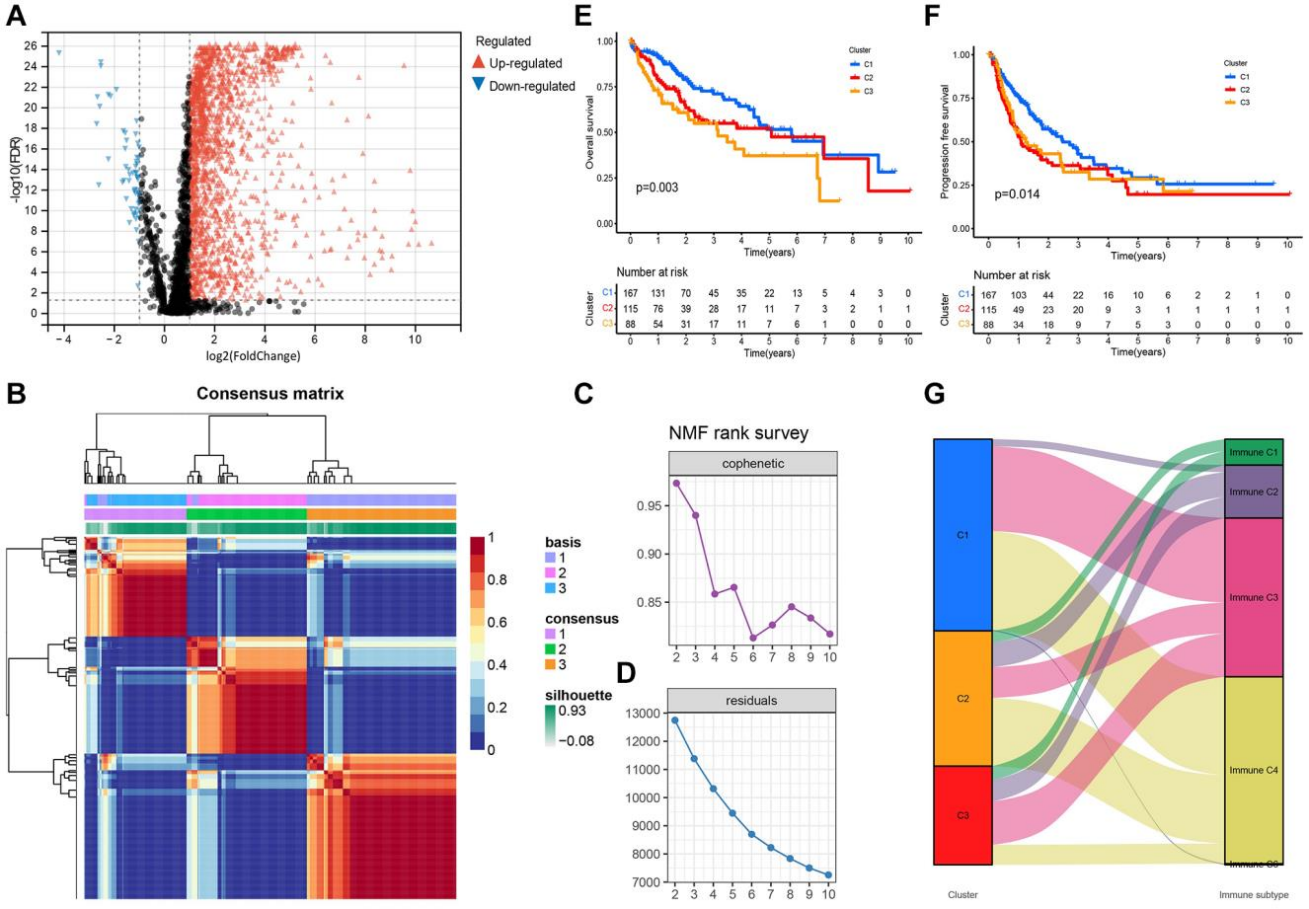
**Differential expression and NMF consensus clustering analysis of TRGs.** (**A**) Volcano plot shows DE-TRGs among 374 patients with HCC and 50 control samples. (**B**) NMF consensus clustering for the k = 3. (**C**) The cophenetic correlation coefficient indicates the stability of the cluster obtained using the NMF algorithm. (**D**) RSS reflects the performance of the model in clustering. (**E**) The KM survival curves show the OS and (**F**) the PFS of patients in the three subtypes. (**G**) The alluvial map shows the distribution of these subtypes in immune subtypes.

**Figure 2:**
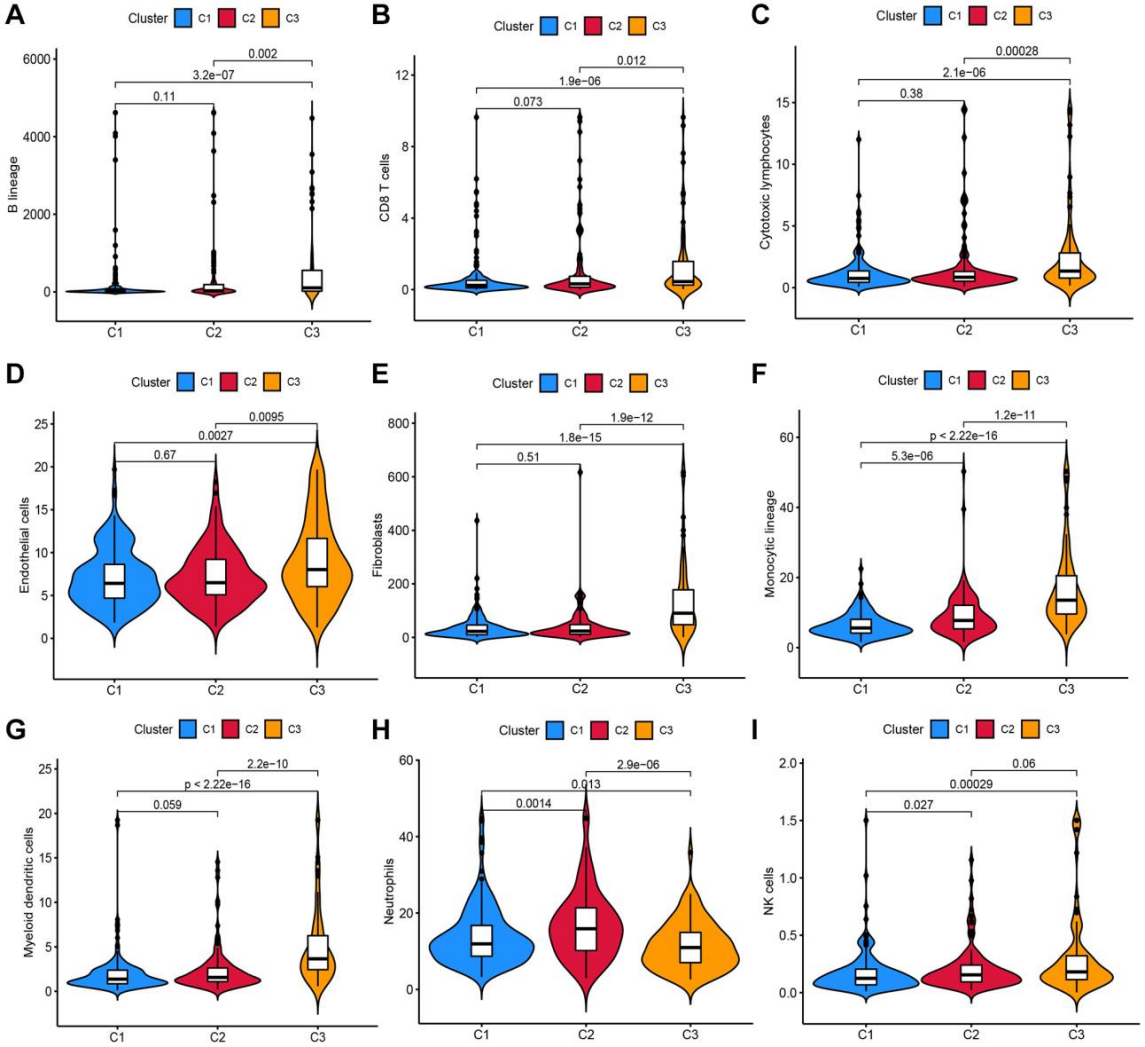
Analysis of the infiltration scores of (**A**) B lineage, (**B**) CD8+ T cells, (**C**) cytotoxic lymphocytes, (**D**) endothelial cells, (**E**) fibroblasts, (**F**) monocytic lineage, (**G**) myeloid DCs, (**H**) neutrophils and (**I**) NK cells in patients in these three molecular subtypes.

A total of 1,717 TRGs were defined as DE-TRGs from 4061 TRGs between 370 (FDR <0.05 and |log2FC|>1, Figure 1A), and UCRA identified 704 prognosis-related TRGs. NMF consensus clustering analysis was performed based on the expression profiles of 704 prognosis-related TRGs. The cophenetic correlation coefficient was significantly reduced at k = 3, which was therefore selected as the optimal number of clusters (Figure 1B–1D). Survival analysis showed that the OS and progression-free survival (PFS) of patients in C2 and C3 was significantly worse compared to C1 (Figure 1E, 1F). Moreover, immune scores of myeloid dendritic cells, B lineage, endothelial cells, cytotoxic lymphocytes, neutrophils, fibroblasts, monocytic lineage, NK cells, and CD8+ T cells were different across C1, C2, and C3 (Figure 2A–2I).

Wound healing (Immune C1), IFN-gamma dominant (Immune C2), inflammatory (Immune C3), lymphocyte depleted (Immune C4), C5 (Immunologically quiet), and TGF-beta dominant (Immune C6) are the six immune subtypes of solid tumors [23]. Hence, the distribution of these immune subtypes in three molecular clusters was analyzed. Specifically, Immune C3 and C4 were the primary immune subtypes in C1, the proportions of Immune C4 and C3 were the highest in C2 and C3, respectively, and Immune C1 was only detected in C2 and C3 (Figure 1G).

### Constructing and validating the RS model containing 13 DE-TRGs

**Figure 3:**
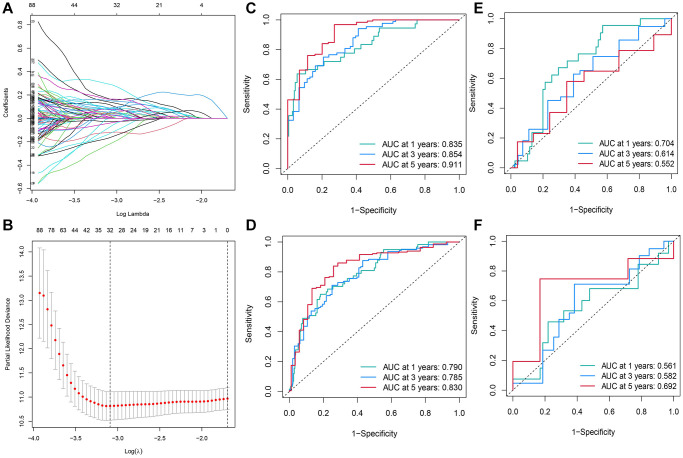
**Construction of the RS model constituting 13 DE-TRGs.** (**A**) The LASSO coefficient profile of the DE-TRGs in patients from the training set. (**B**) The partial likelihood deviance method was used for screening genes. (**C**–**F**) Constructing and validating the RS model with the AUC value for 1-, 3- and 5-year OS of patients with HCC in (**C**) training set; (**D**) entire TCGA cohort; (**E**) validation set; (**F**) GEO cohort.

UCRA and LASSO regression analysis was conducted using the DE-TRGs identified from the training set. Independent variables gradually increased to zero with lower λ (Figure 3A). In addition, 32 genes were mined using the partial likelihood deviance method (Figure 3B). Finally, the RS model was constructed with the 13 TMGs identified by MCRA as follows:

Risk score = (−0.5974 × CAPN3 expression level) + (0.3376 × HAVCR1 expression level) + (0.4161 × GRIN2D expression level) + (0.6536 × BMI1 expression level) + (0.4733 × SLC30A3 expression level) + (0.2293 × GLP1R expression level) + (0.2211 × CSAG3 expression level) + (−0.5401 × NR4A3 expression level) + (−0.8607 × INPP4B expression level) + (0.1339 × ETV4 expression level) + (−0.2406 × MYH4 expression level) + (−0.4394 × ICA1 expression level) + (0.4485 × ST6GALNAC4 expression level.

Next, the AUC value was calculated to assess the RS model in all cohorts. The AUC values for 1-, 3-, and 5-year OS of patients in the training set were 0.835, 0.854, and 0.911, respectively (Figure 3C). In the entire TCGA-LIHC cohort, the AUC values for 1, 3, and 5-year OS of patients were 0.790, 0.785, and 0.830, respectively (Figure 3D). In the TCGA validation cohort (the validation set), the AUC values for 1-, 3-, and 5-year OS of patients were 0.704, 0.614, and 0.552, respectively (Figure 3E). Though the AUC values of the 1- (0.561), 3- (0.614), and 5-year (0.692) OS of patients in the GSE76427 cohort were <0.7, the RS model was still accurate to some extent, especially in predicting the 5-year OS. Such a result might be related to the short median duration of follow-up of patients in this cohort (Figure 3F).

**Figure 4:**
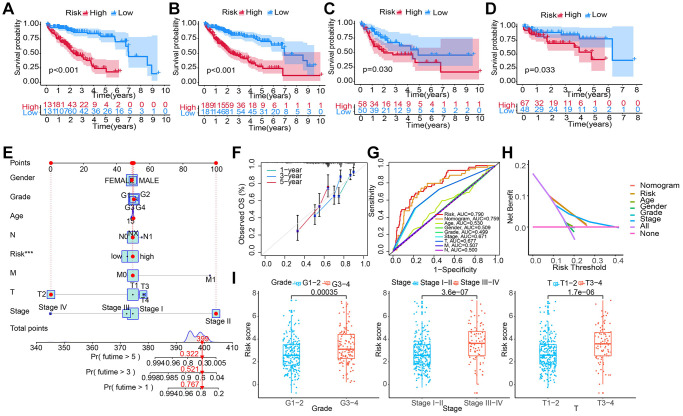
**Constructing and evaluating a nomogram by integrating the RS and clinicopathological features.** (**A**–**D**) Construction and validation of the TRG-RS in patients with HCC in (**A**) training set; (**B**) entire TCGA cohort; (**C**) TCGA testing cohort; (**D**) GEO cohort using the KM survival curves. (**E**) Nomogram predicting the 1-, 3- and 5-year patient’s OS. The points identified on the point scale of each variable are totaled. Finally, beneath the total points, the probability of 1-, 3- or 5-year survival is projected on the scales below. (**F**) CC shows the actually observed and nomogram-predicted 1-, 3- and 5-year OS of patients. (**G**) The ROC curve shows the AUC value of the nomogram, the RS, and clinicopathologic features for predicting the patient’s survival. (**H**) Evaluating the clinical benefit of the nomogram and the RS using DCA. None indicates that all samples were negative and untreated; therefore, the net benefit is zero. All indicates that all samples were positive and treated. The x-axis shows the threshold probability. (**I**) TRG-RS comparison of clinical information of patients with HCC in TCGA-LIHC.

Patients from all HCC cohorts were assigned with a RS and divided into HRG and LRG based on the median RS. The KM survival curves showed that in the training set, the prognosis in the LRG was significantly better than in HRG (Figure 4A). The results from survival analyses on patients in the validation set, the entire TCGA, and the GSE76427 cohort were the same results. In the HRG, the patient’s OS was significantly poor than in LRG (Figure 4B–4D). Additionally, in the TCGA-LIHC cohort, the RS varied in patients with different clinicopathological characteristics and it was positively correlated with high pathological grades and advanced T, TNM stages (Figure 4I).

### Construction of a nomogram by integrating RS and clinicopathological features

We performed UCRA and MCRA to determine the correlation between the OS and clinicopathological features such as age, tumor grade, and stage, gender, T and M stage, as well as RS. The results demonstrated that only the RS (HR: 1.026, 95% CI: 1.014–1.037, *P* < 0.001) could independently predict risk (Table 2). A nomogram integrating clinicopathological features and RS was developed (Figure 4E) to predict 1-, 3-, and 5-year survival. For example, the clinical features of a 50-year-old female with LIHC were T2NxM0 stage II, grade 1, and high RS. Then, final total score of the variables was 399. The AUC values of patients’ 1-, 3-, and 5-year survival rates were 0.767, 0.561, and 0.322, respectively. The CC of the nomogram showed a high consistency between the observed 1-, 3- and 5-year survival rates and the predicted values in the training sets (Figure 4F). In addition, compared to the other clinicopathological characteristics, the AUC value of the RS was higher (Figure 4G). DCA showed that the RS and nomogram could effectively predict the OS compared to all other clinicopathological features (Figure 4H).

**Table 2:** Univariate and multivariate Cox regression for risk score and clinicopathologic features.

**Variables**	**Univariable analysis**			**Multivariable analysis**		
	**HR**	**95% CI**	***p*-value**	**HR**	**95% CI**	***p*-value**
Age	1.011	0.996–1.0258	0.147	−	−	−
Gender	0.765	0.524–1.119	0.167	−	−	−
Grade	1.123	0.872–1.445	0.369	−	−	−
Stage	1.675	1.364–2.0567	8.54E-07	0.958	0.430–2.132	0.915
T	1.655	1.361–2.012	4.48E-07	1.693	0.796–3.600	0.171
M	3.78	1.196–11.946	0.024	1.505	0.439–5.153	0.515
RiskScore	1.024	1.013–1.034	6.71E-06	1.026	1.014–1.0367	5.18E-06

### Comparing the TRG-RS model with other models

**Figure 5:**
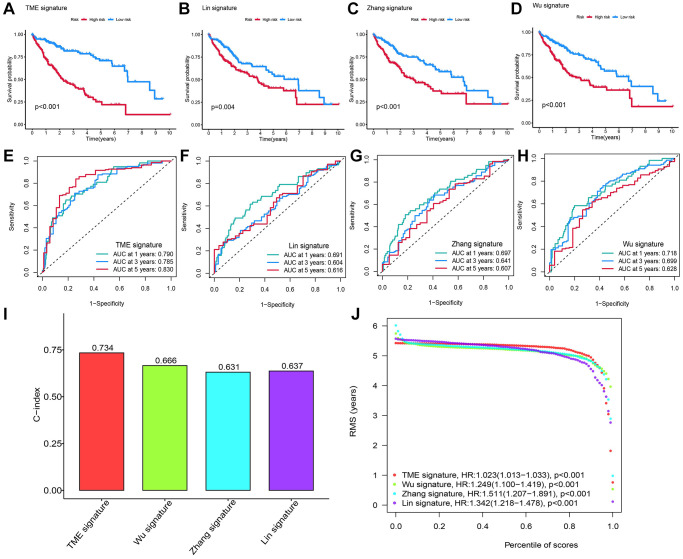
**Comparing the RS model and three previously published models.** (**A**–**D**) The KM survival curves of patients were predicted by our RS model and three previously published models. (**E**–**H**) The ROC curves and (**I**) the C-index of our RS model and three published models and the C-index of our RS model were the highest. (**J**) The RMS time curve of all four prognostic RS models revealed an overlap of 60 months.

We compared our 13-gene signature RS model to other models, including 8-gene signature related to inflammation response (a TME signature) [24], 3 immune-related prognostic genes [14], and 3 tumor doubling time-related immune genes [15]. Firstly, we calculated the RS for all patients using MCRA and plotted the ROC curve using their corresponding genes. Then, the patients were classified by the median RS value into HRG and LRG. A significant survival difference of patients in the two groups was observed. In addition, the prognosis of patients in LRH was better than in HRG (Figure 5A–5D). As shown by the ROC curve, the AUC value of our model in predicting average 1- year prognosis was the highest. The AUC values of our model for predicting 1-, 3-, and 5-year survival were higher compared to the other three models (Figure 5E–5H). The C-index of our model was 0.734, which was higher than other models (Figure 5I). Finally, we used the RMS time to assess the prediction accuracy of our model at different time points, and observed that our model outperformed the other three models when predicting survival time longer than 60 months. These results indicated that our model was more effective and accurate in predicting 5-year OS and survival timer longer than 5 years (Figure 5J).

### Functions of our RS model

**Figure 6:**
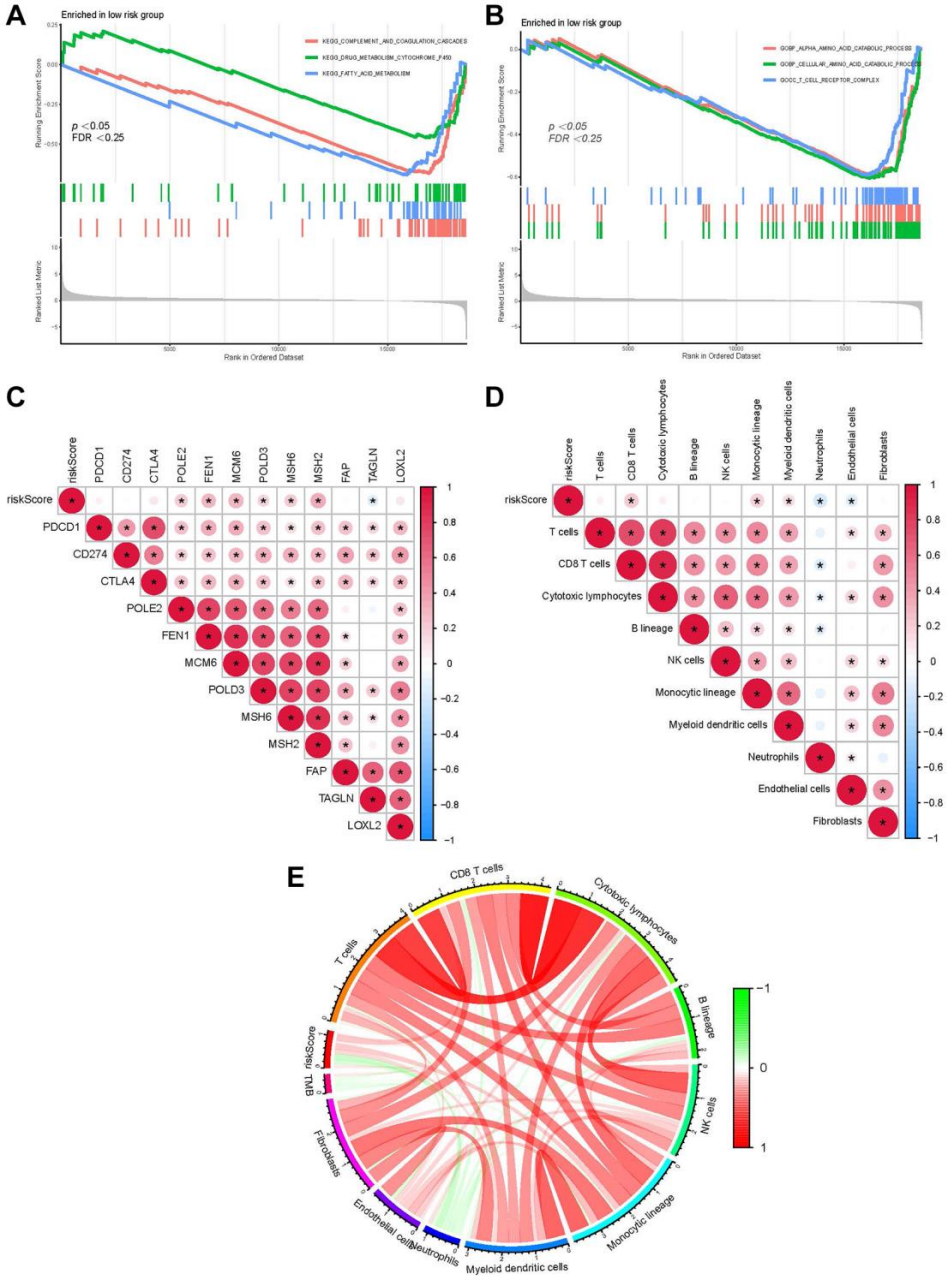
**Function of our RS model.** (**A**) GSEA shows the KEGG pathways enriched in the LRG. (**B**) GO terms enriched in the LRG. (**C**) The correlation between the RS of patients with LIHC and the expression of cancer-related pathway genes. (**D**) The correlation between immune cell score in patients from TCGA-LIHC and the RS. (**E**) Correlation between the RS/TMB and immune cell score. Among them, ^*^usually represents a *p*-value < 0.05. Red indicates a positive correlation, blue indicates a negative correlation, and the shade of the color indicates the strength of the correlation.

Based on the normalized enrichment score and adjusted *p*-value (*q*-value), GSEA was applied to identify enriched pathways in the two groups. The complement and coagulation pathways, fatty acid and cytochrome p450 drug metabolism was enriched by genes in the LRG (Figure 6A). Moreover, GO terms such as the process of cellular amino acid and alpha-amino acid catabolism and the T cell receptor complex were enriched genes in the LRG (Figure 6B). The correlation between the RS and target genes was analyzed according to the expression of genes related to classical cancer-related pathways including fatty acid metabolism, DNA replication, and cell cycle. DNA replication-related genes (*MCM6, MSH2*, and *MSH6*) were positively correlated with the RS (Figure 6C). Additionally, immune cell infiltration in all patients from TCGA-LIHC was analyzed. The RS showed a positive relationship with CD8+ T cells, monocytic lineage, and myeloid DCs (Figure 6D). The correlation between the RS/tumor mutation burden (TMB) and infiltrating immune cells was analyzed using an MCP counter, and the results are presented in Figure 6E.

### Survival prediction for patients with different clinicopathological characteristics using the RS

**Figure 7:**
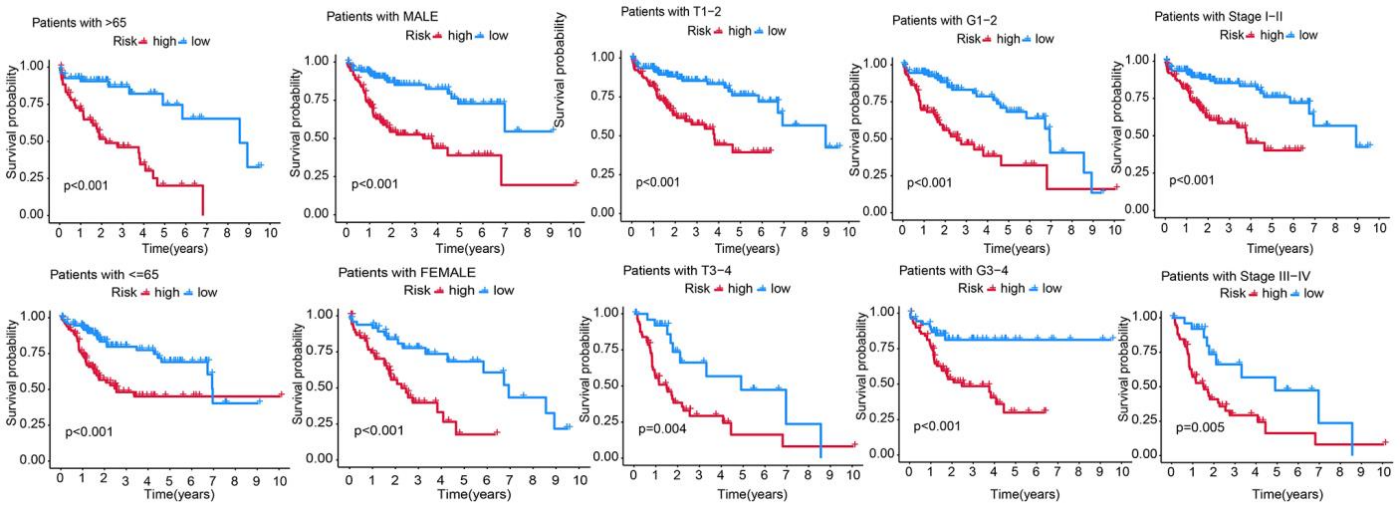
Predicting the survival of patients with different clinicopathological features, including age (age >65 and ≤65), gender, T stage (T1-2, T3-4), AJCC stage (stage I–II and stage III–IV), and pathological grade (G1-2 and G3-4) using the RS model.

We predicted the survival of patients with different clinical traits using the RS model. Compared to those in HRG, the KM survival showed better prognostic outcomes of patients with age >65 and ≤65, pathological grade (G1-2 and G3-4), gender, stage I–II, and III–IV in the LRG (Figure 7).

## DISCUSSION

HCC is a typical inflammation-induced cancer. A complex interaction between TMEs is a prerequisite for HCC development [25]. Comprehensive and systematic profiling of these complex interactions in heterogeneous tumor samples could help effectively identify therapeutic agents and biomarkers [26]. This study analyzed TME-related genes to explore the molecular heterogeneity of HCC. Targeting a single molecule involved in the interactions between tumor and TME is unlikely to accurately predict the prognosis of HCC [27]. Hence, we developed an RS model based on multiple TRGs to predict patients’ prognosis and determine the biological effects of HCC.

Classifying molecular subtypes using TME-associated genes represents a key advance in understanding the heterogeneity of HCC, and these isoforms could reflect TME characteristics, different stages of tumor progression, immune evasion capacity and response to therapy [28]. Zhang et al. revealed three unique HCC subtypes of immunoactive, immunodeficient, and immunosuppressive TME using CyTOF [29]. In this study, HCC patients were classified into different subtypes based on the transcriptomic characteristics of TRGs. We identified 4,061 TRGs, of which 704 prognosis-related DE-TRGs were screened by differential expression analysis and UCRA. Based on the expression matrix of these DE-TRGs, we divided patients with HCC into C1, C2, and C3 molecular subtypes. Specifically, patients in subtype C1 exhibited a more favorable prognosis than those in subtypes C2 and C3. We then employed CRA and LASSO regression analysis to construct an RS model based on these 13 TRGs.

The effectiveness of the RS model in predicting the patient’s prognosis was analyzed. UCRA and MCRA results revealed that only the RS was an independent risk (not clinicopathological features) for patients with HCC from TCGA-cohort. Furthermore, the reliability of our model was validated according to the CC, AUC value, C-index, and DCA. Furthermore, our RS models outperformed the other three previously published models in predicting HCC prognosis. These results confirmed the reliability of our prognostic model.

We also analyzed the potential pathways enriched by our RS model. Dysregulated pathways, such as fatty acid metabolism [30], DNA replication [31], and cell cycle [32], are involved during tumorigenesis. Correlation analysis between the RS and target genes also showed a positive relationship between DNA replication-related genes and the RS. CD8+ T cells were positively related to the RS. CD8+T cells primarily mediate anti-tumor immune responses [33]. A previous study showed better survival outcomes of cancer patients with high CD8+ T cell levels [34]. However, patients with a high RS associated with CD8 +T cell score had poor prognosis, which might be related to tumor cell escape from immune surveillance mediated by CD8 +T cells.

In summary, we identified three molecular HCC subtypes based on TRGs using NMF. An RS model was established using these 13 TRGs and the prognostic value and application of the RS model was verified in clinical settings. However, additional experimental validation is needed to confirm the underlying mechanism of TRGs of the RS model in HCC.
